# COVID-19 symptom severity predicts neutralizing antibody activity in a community-based serological study

**DOI:** 10.1038/s41598-022-15791-6

**Published:** 2022-07-18

**Authors:** Amelia Sancilio, Joshua M. Schrock, Alexis R. Demonbreun, Richard T. D’Aquila, Brian Mustanski, Lauren A. Vaught, Nina L. Reiser, Matt P. Velez, Ryan R. Hsieh, Daniel T. Ryan, Rana Saber, Elizabeth M. McNally, Thomas W. McDade

**Affiliations:** 1 Institute for Policy Research, Northwestern University, Northwestern University, 1810 Hinman Ave, Evanston, IL 60208 USA; 2 Institute for Sexual and Gender Minority Health and Wellbeing, Northwestern University, Evanston, USA; 3 Department of Medical Social Sciences, Northwestern University Feinberg School of Medicine, Chicago, USA; 4 Department of Anthropology, Northwestern University, Evanston, USA; 5 Center for Genetic Medicine, Northwestern University, Evanston, USA; 6 Department of Pharmacology, Northwestern University Feinberg School of Medicine, Chicago, USA; 7 Division of Infectious Diseases, Department of Medicine, Northwestern University Feinberg School of Medicine, Chicago, USA; 8 Division of Cardiology, Department of Medicine, Northwestern University Feinberg School of Medicine, Chicago, USA; 9 Department of Biochemistry and Molecular Genetics, Northwestern University, Evanston, USA

**Keywords:** Infection, Immunology, Antibodies

## Abstract

Serological testing for SARS-CoV-2 IgG antibodies is used to assess their presence in blood samples from exposed individuals and provides a measure of the magnitude of immune response to infection. The measurement of neutralizing antibodies (NAbs) in particular provides information about the severity of prior infection and level of protective immunity against re-infection. Much of the work investigating the association between prior infection severity and NAb levels has been conducted among clinical populations, and less is known about this relationship in the general population. Accordingly, we utilize data from a large (n = 790) community-based cohort of unvaccinated, seropositive participants. We analyzed the association between NAb response, measured via surrogate virus neutralization assay, with patterns of symptoms and household exposure. Our results indicate no detectable NAb activity in 63.8% of the seropositive participants (n = 504). Those with detectable NAb levels demonstrated a positive relationship between NAb activity and both self-reported previous symptom severity and household exposure. These findings are significant in light of recent concerns about degree of protective immunity conferred by prior infection or vaccination, and we highlight the value of community-based research for investigating variation in immune response.

## Introduction

As of July 22, 2021, more than 190 million people worldwide have been infected by the severe acute respiratory syndrome coronavirus 2 (SARS-CoV-2), which causes coronavirus disease 2019 (COVID-19)^1,2^. Response to infection with SARS-CoV-2 can be highly variable, including differential degrees of symptom severity, hospitalization, and immune response. Serological testing has been a vital tool in tracking SARS-CoV-2 infection across communities, as it is used to detect the presence of antibodies to SARS-CoV-2 in blood samples from exposed individuals, while also providing a measure of the magnitude of immune response to infection^3,4^.

Serological testing facilitates assessment of exposure and immune response via laboratory measurement of antibodies that impede infection by SARS-CoV-2. Some, but not all, antibodies directed against the spike protein of SARS-CoV-2 can neutralize virus infectivity in laboratory assays. These neutralizing antibodies (NAbs) bind to the SARS-CoV-2 surface spike protein on the virion surface, the part of the virus that engages the human angiotensin-converting enzyme 2 (ACE2) receptor in order to gain entry into host cells^5,6^. NAbs are effective at inhibiting this initial interaction, as well as downstream events, and thereby preventing viral entry into cells^5^. Following infection, NAbs have been shown to persist for many months, albeit with decreasing levels detectable in the blood^7,8^.

Because of their key role in hindering viral entry, it is expected that neutralizing antibody activity can provide information about severity of prior infection and the level of protective immunity against re-infection. This expectation is supported by results indicating that NAbs appeared earlier and at higher levels in patients who experienced severe or moderate infections, when compared with patients who experienced mild or asymptomatic illness^9^. Similarly, COVID-19 patients who were treated in an intensive care unit demonstrated significantly higher peak neutralizing antibody titers compared with those who were not^10,11^. Further, higher neutralizing antibody levels among vaccinated individuals have been shown to predict a lower likelihood of SARS-CoV-2 breakthrough infections^11^. These results point to a consistent relationship between disease severity and level of NAb activity in clinical populations.

Less is known about this relationship in the general population. Increasing our understanding of variation in NAb levels across severity of infection in the community is particularly critical as many people remain hesitant to receive the SARS-CoV-2 vaccine, based on beliefs that any prior exposure will provide adequate protection against re-infection by the virus^12^. We utilized data from a large community-based cohort in Chicago in order to investigate these dynamics in a non-clinical population that includes people who did require treatment or even know they had been infected with SARS-CoV-2. Because the majority of COVID-19 cases are asymptomatic or mild^4,13^, our community-based sample adds a clearer picture of the nature of the association between disease severity and the production of NAbs, which likely provides at least partial protection against re-infection in the broader population of all previously infected and unvaccinated persons.

Previous work by our team using data from a community-based observational study in Chicago has shown that those who reported more symptoms of infection in months prior to antibody quantitation had higher concentrations of immunoglobulin G (IgG) antibodies directed against SARS-CoV-2 spike receptor binding domain (RBD)^14,15^. Previous work also indicated that household exposure to the virus, which is likely greater or more prolonged than exposure outside the home, is associated with both greater disease severity and higher antibody concentrations^15^. However, it is not yet known whether there is a similar association between the level of NAb activity and either COVID-19 symptom severity or household exposure history in a non-clinical population.

Here, we analyze the association of magnitude of NAb response, using a surrogate virus neutralization assay, with patterns of symptoms and household exposure history among unvaccinated, seropositive participants. Our results indicate that the majority of individuals who tested seropositive for prior COVID-19 infection had no detectable neutralization activity. In individuals who had detectable levels of neutralizing antibodies, we report a positive association between COVID-19 symptoms and neutralization activity. We also report higher neutralization activity in individuals living with household members who reported symptoms or diagnosis of COVID-19.

## Methods

### Study design

A large community-based sample of adults living within the Chicago, IL metropolitan area was recruited to participate in a study called Screening for Coronavirus Antibodies in Neighborhoods (SCAN). Samples for this analysis were collected between June 24 and November 11, 2020, prior to availability of SARS-CoV-2 vaccination in the area^4^. The study included a total of 4463 adults at this time point.

Participation was facilitated by a web-based, “no contact” protocol, which allowed individuals to provide information and dried blood spot (DBS) samples outside of a clinical setting. Participants were recruited via advertisements in social media, emails, print flyers, newspapers, participant registries, participant referrals, community outreach, and local press. Recruitment was conducted in neighborhoods throughout the Chicago metropolitan area and at the Northwestern University Feinberg School of Medicine (FSM) in Chicago. To ensure racial and gender diversity and representation within the sample, we carried out stratified random sampling to adaptively match enrollment of white participants and women (groups that were more likely to complete the screener) to enrollment of non-white participants and men.

Study data were collected and managed using REDCap electronic data capture tools hosted at Northwestern University^16,17^. All research activities were implemented under protocols approved by the institutional review board at Northwestern University (#STU00212457 and #STU00212472). Written informed consent was received from all individuals prior to participation in this study, and all methods were performed in accordance with relevant guidelines and regulations.

Participants provided information about COVID-19 testing, diagnoses, and symptoms experienced after March 1, 2020. Other variables included sex (based on assignment at birth), self-identified racial/ethnic identity, pre-existing chronic medical conditions (having one or more of the following: kidney disease, lung disease, diabetes mellitus, cardiovascular disease, or body mass index > 30 kg/m^2^), smoking, number of individuals in their households, and whether any cohabitants had been diagnosed with COVID-19 or had symptoms of COVID-19.

After completing the survey, a kit containing materials for collecting a finger stick DBS sample was mailed to participants’ homes or made available for pick-up for onsite medical school participants. The kit included instructions for self-collection, as well as information for online video instructions. Following collection, samples were mailed to the lab for analysis using pre-stamped envelopes.

There was no difference in the number of days between the timing of reported symptoms and blood collection across seronegative and seropositive participants, and between participants who did and did not have detectable neutralization activity. All study participants were comparable in the number of days since the start of the pandemic (defined as March 1, 2020) and the timing of blood collection (Supplementary Table 1).

Analyses presented here focus on the 17.7% (n = 790) of the 4463 total participants that tested seropositive for prior infection based on the presence of IgG antibodies against the RBD of SARS-CoV-2^18^. IgG antibodies were quantified using an enzyme-linked immunosorbent assay (ELISA) that has received emergency use authorization from the United States Food and Drug Administration (COVID-SeroKlir, Kantaro Biosciences). This assay was adapted and validated for use with DBS samples^18^.

### Evaluation of symptoms

Prior to DBS collection, participants indicated whether they had experienced any of the following eleven symptoms potentially associated with COVID-19: headache; fatigue or excessive sleepiness; sore throat; cough; muscle or body aches; runny nose; fever or chills; diarrhea, nausea, or vomiting; shortness of breath; loss of sense of smell or taste; and itchy eyes. In a previous study, we identified a cluster of eight of these symptoms that were associated with higher SARS-CoV-2 IgG concentrations: headache; fatigue or excessive sleepiness; cough; muscle or body aches; fever or chills; diarrhea, nausea, or vomiting; shortness of breath; loss of sense of smell or taste^14,15^. The symptoms not included in the cluster of eight are itchy eyes; runny nose; and sore throat.

A “COVID-19 symptom severity score” for each participant was created following methods described previously (Schrock et al.^15^). Briefly, each of the eight symptoms associated with higher SARS-CoV-2 IgG antibody levels was weighted by the regression coefficient that resulted from a bivariate model that included the symptom as the independent variable and log10-transformed SARS-CoV-2 IgG concentration as the dependent variable. For the symptom score, our goal was to establish the independent association between each individual symptom and SARS-CoV-2 IgG antibody levels—which was facilitated by using bivariate models (that did not include other symptoms). Because participants reported distinct combinations of symptoms (i.e., reported some and not others), we did not seek to create scores that included the correlation between symptoms or their mutual influence, as doing so might under-or over-estimate the significance of the individual symptom. The resulting symptom weights are: fever = 0.22, cough = 0.13, shortness of breath = 0.20, headache = 0.09, muscle or body aches = 0.19, fatigue or excessive sleepiness = 0.13, diarrhea/nausea/vomiting = 0.17, loss of taste/smell = 0.32^15^. This composite variable indicated how severely each participant experienced symptoms associated with COVID-19 infection.

### Measurement of SARS CoV-2 neutralizing antibody activity

All samples were analyzed in duplicate for anti-spike SARS-CoV-2 neutralizing antibodies with a commercially available surrogate virus neutralization test (sVNT) protocol (Meso Scale Diagnostics V-PLEX SARS-CoV-2 Panel 2 Kit; K15386U-2). This method, which can be implemented with immunoassay techniques, contrasts with other conventional methods for measuring neutralizing activity that require the presence of live virus and specialized laboratory containment facilities. Excellent concordance between results of these types of assays of NAb activity has been reported^19^. To replicate the interaction that occurs between the virus and a host, the sVNT method incubates the blood samples with purified versions of viral spike protein and human ACE2 receptor. The neutralizing antibodies present in the samples prevent the spike protein from binding to the ACE2 receptor, and the competitive immunoassay quantifies the degree of inhibition in order to measure NAbs levels.

The sVNT method was adapted and validated to measure neutralizing antibodies in DBS samples described here^20^. Based on these validation results, the threshold for determining the presence of surrogate neutralization activity was set at 13.2% or higher^20^.

### Statistical analyses

Patterns of association between symptoms and the presence of NAb were established using summary statistics and multiple linear regression analyses. Due to the skewed distribution of NAb values, median values are presented as descriptive statistics and log10-transformed values were used for regression analyses. Covariates in the multiple linear regression analyses included age, race/ethnicity, sex assigned at birth, and chronic pre-existing conditions. All data analyses were conducted using R (version 4.0.4) in RStudio Version 1.4.1106^21^.

## Results

Table 1 provides demographic statistics of participant characteristics for the whole seropositive sample population, as well as demographic statistics for participants who did and did not have detectable neutralizing antibody levels. Of the 790 seropositive participants, mean age was 38.6 years (range 18–81, SD = 13.2) and 55.1% reported their birth sex as female. Hospitalization was reported by 11.0% of seropositive participants, and 4.9% of participants reported receiving a positive PCR swab diagnosis of SARS-CoV-2 infection. No neutralization activity was detected in 63.8% of the seropositive participants (n = 504). Among the 36.2% of seropositive participants (n = 286) with neutralization activity, the median surrogate neutralization level was 23.5% (interquartile range 16.9–40.6%).

**Table 1:** Demographic statistics of seropositive sample population. N = 790.

	Total sample (n = 790)	No neutralizing activity (n = 504)	Neutralizing activity (n = 286)
Mean age (IQR^a^)	38.6 (28–47)	37.8 (29–49)	40.0 (27–45)
**Gender**			
Female	435 (55.1%)	289 (57.3%)	146 (51.0%)
**Race/ethnicity**			
Asian	186	138	48
Black, non-Latinx	67	39	28
Latinx	168	92	76
Other	20	8	12
White, non-Latinx	349	227	122
**Pre-existing chronic medical conditions**			
Yes	182 (23.0%)	105 (20.0%)	77 (27.1%)
**Smoking**			
Yes	52 (6.6%)	26 (5.6%)	26 (9.1%)
**Positive PCR?**			
Yes	31 (4.9%)	6 (1.2%)	25 (8.7%)
**Whether any cohabitants symptoms of COVID-19**			
Yes	277 (35.1%)	149 (29.5%)	128 (44.8%)
**Whether any cohabitants had been diagnosed with COVID-19**			
Yes	91 (11.5%)	24 (4.8%)	67 (23.4%)

^a^Inter-quartile range.

### Neutralization activity and total number of reported symptoms

Of the seropositive participants included in these analyses, 67.7% of participants reported experiencing one or more of eleven COVID-19 symptoms listed above since March 1, 2020. Notably 32.3% reported no prior symptoms.

**Figure 1:**
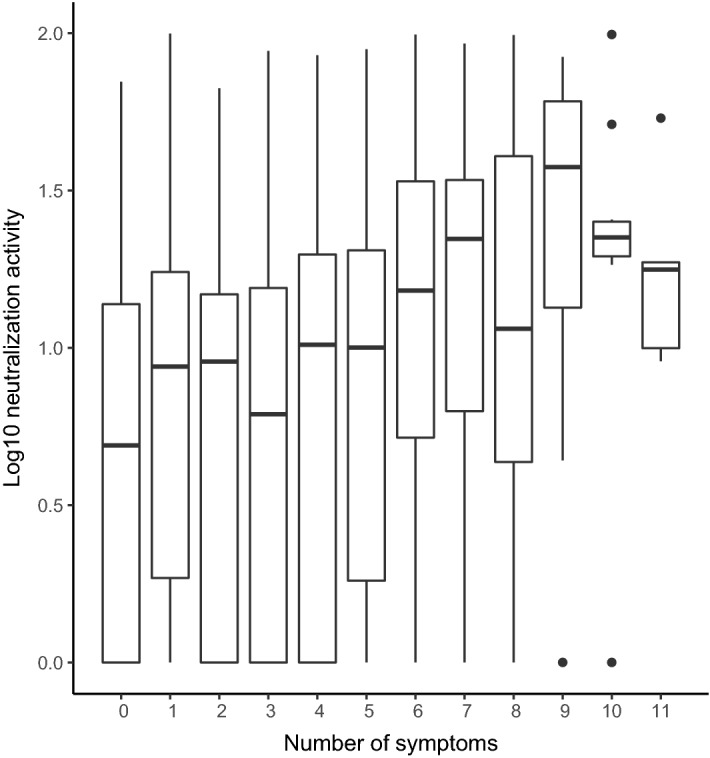
Percent inhibition of Spike-ACE2 binding in association with number of reported symptoms in seropositive individuals. Boxplot indicating mean, interquartile range, and outlier measurements of log10-corrected surrogate neutralization levels for seropositive participants who reported 0–11 symptoms of COVID-19 infection^21^. N = 790.

Individuals with surrogate neutralization activity detected in this assay were more likely to report any symptoms than individuals without detectable NAb activity (Table 2). These individuals also reported a higher total frequency of symptoms than individuals without detectable neutralization activity (Wilcoxon rank-sum Z = − 6.01, p < 0.01). The median number of symptoms reported by participants with neutralizing antibody activity was three (IQR = (0–6), while participants without neutralization activity experienced a median of two symptoms (IQR = 0–2). Consistently, a linear regression model, controlling for the covariates listed above, demonstrated a positive linear association between total number of reported COVID-19 symptoms and surrogate neutralization levels (β = 0.26; R^2^ = 0.12; p < 0.001) (Table 3, Model B; Fig. 1).

**Table 2:** Prevalence of reported symptoms of SARS-CoV-2 infection within the total seropositive sample, proportion of individuals with and without neutralizing antibody activity, and magnitude of surrogate neutralization activity by symptom for seropositive individuals.

	Total sample (%) (n = 790)	No neutralizing activity (%) (n = 504)	Neutralizing activity (%) (n = 286)	Median NAb (%)	25th %ile	75th %ile
Headache	41.27	35.52	51.40***	11.29	1.80	24.48
Fatigue	31.77	27.18	39.86***	11.09	2.23	29.75
Sore throat	28.48	28.37	28.67	9.26	1.74	18.37
Cough	27.97	21.83	38.81***	13.22	3.24	26.59
Muscle or body aches	27.59	21.03	39.16***	13.98	5.09	32.97
Runny nose	27.59	27.98	26.92	8.06	0.41	18.97
Fever	23.04	14.68	37.76***	18.37	6.33	39.12
Diarrhea	17.47	12.90	25.52***	13.98	2.65	36.6
Shortness of breath	13.29	8.33	22.03***	19.50	7.57	35.87
Loss of sense of smell or taste	13.80	5.36	28.67***	26.69	13.23	53.98
Itchy eyes	12.78	12.70	12.94	8.98	0.2	18.7
No symptoms	32.28	36.71	24.48***	4.91	0	13.95

N = 790.

Pearson chi-square test of independence: ***p < 0.001.

**Table 3:** Coefficients, with standard errors in parentheses, from linear regression models with log_10_ surrogate neutralization levels as outcome variable.

	Outcome variable: log_10_ surrogate neutralization levels (%)			
	Model A	Model B	Model C	Model D
Age	0.072* (0.036)	0.074* (0.035)	0.065 (0.034)	0.061 (0.036)
Assigned female at birth	− 0.197** (0.070)	− 0.230*** (0.068)	− 0.238*** (0.066)	− 0.214** (0.070)
**Race/ethnicity (ref = white)**				
Hispanic/Latinx	0.389** (0.093)	0.302*** (0.090)	0.256 ** (0.088)	0.356*** (0.093)
Black	0.310* (0.132)	0.317* (0.128)	0.261* (0.125)	0.315** (0.131)
Asian	− 0.081 (0.091)	− 0.048 (0.088)	− 0.051 (0.085)	− 0.068 (0.090)
Other	0.425 (0.224)	0.361* (0.217)	0.302 (0.211)	0.381 (0.223)
Chronic pre-existing conditions	0.188* (0.086)	0.139* (0.083)	0.116 (0.081)	0.185* (0.085)
Number of symptoms		0.259*** (0.034)		
Symptom severity score			0.338*** (0.034)	
Household member diagnosed with COVID-19 or who reported COVID-19 symptoms				0.273*** (0.073)
Intercept	− 0.036 (0.065)	0.005 (0.063)	0.032 (0.062)	− 0.117 (0.068)
Adjusted R^2^	0.054***	0.118***	0.162***	0.069***

N = 790.

*p < 0.05; **p < 0.01; ***p < 0.001.

In addition to a greater likelihood of experiencing more of any of the COVID-19, individuals with neutralization activity were more likely to have reported one of the eight symptoms demonstrated to be associated with higher likelihood of COVID-19 infection in our prior study^15^: headache, fatigue or excessive sleepiness, cough, muscle or body aches, fever, diarrhea, shortness of breath, and loss of sense of smell or taste (Table 2).

### Neutralization activity and symptom severity score

Seropositive participants (n = 790) had a mean symptom severity score of 0.32 (range 0.00–1.45, SD = 0.39). COVID-19 symptom severity scores were higher among participants with detectable NAb activity than in participants with no neutralization activity (Wilcoxon rank-sum Z = 10.8; p < 0.001). The median symptom severity score for participants with neutralization activity was 1.16, and median score for participants without detectable neutralization activity was 0.30. A linear regression model adjusted for the same covariates demonstrated a positive linear association between surrogate neutralization levels and COVID-19 symptom severity score (β = 0.34; R^2^ = 0.16; p < 0.001) (Table 3, Model C).

### Neutralization activity and household contacts

Individuals with neutralization activity were more likely to report a household member who had been diagnosed with COVID-19 than participants without detectable levels of NAb activity (23.4% vs. 4.8%; χ^2^ = 17.83; df = 1; p < 0.001). Similarly, participants with neutralization activity were also more likely to have a household member who reported one of the 11 symptoms potentially indicative of COVID-19 (44.8% vs. 29.5%; χ^2^ = 18.68; df = 1; p < 0.001). A regression model adjusted for age, birth sex, race, and preexisting chronic medical conditions demonstrated that living with someone previously diagnosed with COVID-19 or having a household member with symptoms potentially indicative of COVID-19 was positively associated with levels of surrogate neutralization activity (β = 0.27; R^2^ = 0.07; p < 0.0001) (Table 3, Model D). When the same model as above was run with the addition of the quantitative symptom severity score, living with someone previously diagnosed with COVID-19 or having a household member with reported symptoms potentially indicative of COVID-19 was no longer significantly positively associated with levels of surrogate neutralization activity (β = − 0.01, R^2^ = 0.16; p > 0.05). Symptom severity score of the study participant remained positively associated with surrogate neutralization levels in this model (β = − 0.34, p < 0.0001).

## Discussion

Our results indicate a positive relationship between neutralizing antibody activity and both self-reported previous symptom severity and household exposure to the virus, in a community-based sample of unvaccinated, seropositive persons. We also found no detectable surrogate neutralization activity in the majority of seropositive individuals, indicating that lightly symptomatic or asymptomatic SARS-CoV-2 infection does not elicit a strong NAb response.

These results enhance our understanding of the variation in NAb levels across individuals who have different responses to exposure to SARS-CoV-2. These results are particularly significant in light of recent concerns about the persistence of both total antibodies and NAbs to SARS-CoV-2 following infection, particularly in those with mild or asymptomatic cases, and the degree of protective immunity conferred by prior natural infection^22–24^. Our results are consistent with findings from clinical populations that indicate that more severe symptomatic cases of COVID-19 are associated with higher NAb levels, suggesting that these cases are more likely to provide increased and longer-lasting protective immunity following infection^9,25^.

However, our results also highlight that the majority of seropositive individuals in this community-based study did not exhibit neutralization activity. These findings are consistent with other studies conducted with non-clinical populations that have reported a low proportion of seropositive individuals with detectable NAbs^26^. Results such as these support previous findings indicating that prior SARS-CoV-2 infection, especially when mild or asymptomatic, may not be a reliable indicator of ‘natural immunity’ from reinfection, and consistent with the expectation that many previously infected persons are likely to have a relatively low level of protection from reinfection^9,25^. These results are consistent with our previous findings that two doses of the mRNA vaccine were required for individuals who had mild/asymptomatic seropositive cases to attain a level of surrogate neutralizing antibody response comparable to individuals who had previously been diagnosed with COVID-19^27^. Taken together, these findings suggest that natural infection—particularly mild/asymptomatic seropositive cases—are most likely to provide a level of immune protection comparable to one dose of mRNA vaccine^28,29^. Further, our results indicate that, to clinically assess the potential protection conferred from previous infection, it will be helpful to assess symptom severity and method of exposure to the virus, as a surrogate for NAb activity.

Our findings are also relevant to questions concerning the degree of immune protection experienced by individuals who have received a COVID-19 vaccination following SARS-CoV-2 infection. Individuals with previous symptomatic or asymptomatic COVID-19 infection have been shown to have higher neutralizing antibody titer responses to a single dose of mRNA vaccine than those who were not previously infected^29,30^. In addition, completion of two mRNA vaccine doses was reported to elicit a broader NAb response better covering all variants of concern than did full vaccination of previously uninfected persons^31^. However, it is not yet known whether previously infected individuals with varying levels of disease severity differ in their antibody responses that may impact vaccine effectiveness, potential for onward transmission, including for more transmissible Delta variants and other variants of concern that may emerge in the future, after the full course of mRNA vaccinations. Because samples collection for this study predated wide-scale vaccination efforts and the emergence of the Delta variant, we are not able to test these questions directly. Nevertheless, the results of our study highlight the value of community-based research including the full spectrum of SARS-CoV-2 infection for investigating variation in immune response across infected individuals with differential symptomatology. Continuing to examine these dynamics with attention to symptom severity, as well as vaccination history and vaccination responses, can further inform public health strategies to control impacts of SARS-CoV-2 infection.

## Supplementary Information

Supplementary Information.

## Data Availability

The data that analyzed in the current study are available from the corresponding author, AS, upon reasonable request.
