# Supplementary material for: COVID-19 symptom severity predicts neutralizing antibody activity in a community-based serological study
Source: Sci Rep. 2022 Jul 18;12:12269. doi: 10.1038/s41598-022-15791-6 (PMC9293881; doi:10.1038/s41598-022-15791-6)
Supplement: Supplementary file 1 — Supplementary Information. [file 41598_2022_15791_MOESM1_ESM.docx]

**Supplementary Table 1.** Mean number of days between date of sample collection, start of pandemic (defined as March 1, 2020), and date of symptom onset for the total sample of seropositive participants and for individuals with and without neutralizing antibody activity. Total N = 790.

|  | Total sample  (n=790) | No neutralizing activity  (n=504) | Neutralizing activity  (n=286) |
| --- | --- | --- | --- |
| Days between start of pandemic and sample collection | 193.69 | 190.43 | 199.56 |
| Days between symptom onset and sample collection | 131.05 | 127.58 | 135.99 |
